# Pacing in posttraumatic stress disorder therapy: A meta‐regression of treatment frequency and completion rates

**DOI:** 10.1002/jts.70111

**Published:** 2026-07-26

**Authors:** David M. Horton, Gan Shi, Matthew A. Davis, Peter P. Grau, Amy M. Kilbourne

**Affiliations:** ^1^ Department of Learning Health Sciences University of Michigan Medical School Ann Arbor Michigan USA; ^2^ University of Michigan School of Nursing Ann Arbor Michigan USA; ^3^ Office of Research and Development Veterans Health Administration, U.S. Department of Veterans Affairs Washington, DC USA; ^4^ Health Services Research and Development Veterans Affairs Center for Clinical Management Research Ann Arbor Michigan USA; ^5^ Department of Psychiatry University of Michigan Medical School Ann Arbor Michigan USA

## Abstract

The present study utilized posttraumatic stress disorder (PTSD) clinical trials repository data to analyze associations between treatment frequency (i.e., weekly or more than weekly) and completion outcomes across cognitive processing therapy (CPT), prolonged exposure (PE), and eye movement desensitization and reprocessing (EMDR). Covariates included treatment type, interactions between treatment type and treatment frequency, total number of treatment sessions, mean age, and study‐level risk of bias. All studies that met the eligibility criteria were included with CPT, PE, and EMDR in a secondary analysis to evaluate patterns in operationalizing treatment completion. A total of 84 study arms (*k* = 66 studies) were included in the primary analysis. Compared to CPT, patients had slightly lower odds of completing PE, odds ratio (*OR*) = 0.73, 95% confidence interval (CI) [0.62, 0.86], but were twice as likely to complete EMDR, *OR* = 2.44, 95% CI [1.82, 3.27]. Increasing the frequency of PE doubled the odds of completion compared to CPT, *OR* = 2.15, 95% CI [1.55, 2.99]. A Frequency × Treatment interaction signal was nonsignificant for EMDR, *OR* = 1.61, 95% CI [0.96, 2.71]. Secondary outcomes revealed substantial heterogeneity in definitions of treatment completion, with only one third of all CPT, PE, and EMDR trial arms explicitly defining treatment completion as completing all treatment sessions. Future research should expand these findings to other modalities, as well as standardize operationalizations of treatment completion.

Trauma‐focused therapies for posttraumatic stress disorder (PTSD), such as cognitive processing therapy (CPT; Resick et al., 2024), prolonged exposure (PE; Foa et al., 2019), and eye movement desensitization and reprocessing (EMDR; Shapiro, 2018) have demonstrated statistically and clinically significant improvements in PTSD (Asmundson et al., 2018; Kline et al., 2018), with robust evidence for long‐term efficacy (Kline et al., 2018). Such promising findings led to international guidelines from the U.S. Department of Veterans Affairs (VA) and the International Society for Traumatic Stress Studies (ISTSS) classifying PE, CPT, and EMDR as first‐line treatments for PTSD (ISTSS, 2019; VA/Department of Defense [DoD], 2023). Despite their demonstrated efficacy, however, approximately 1 in 5 individuals discontinue PE or CPT before a full dose has been administered (Lewis et al., 2020; Simmons et al., 2021; Varker et al., 2021). These dropout rates, while comparable to rates of psychotherapy dropout in other conditions (Cooper & Conklin, 2015; Swift & Greenberg, 2014), are a major concern in the current PTSD treatment landscape.

Rates of treatment dropout for trauma‐focused treatments have risen as high as 51% in pragmatic‐effectiveness trials (Schnurr et al., 2022), with a weighted dropout rate of 25.6% across all PTSD interventions, among military populations (Penix‐Smith & Swift, 2025). Concerns of noncompletion are elevated by the potential for worsened symptom expression after dropping out of treatment (Kehle‐Forbes et al., 2022). Dropout is a concern both in military and community populations (Varker et al., 2021), with rates even worse in clinical practice (Najavits, 2015). Meta‐analytic research investigating reasons for treatment dropout has been limited by the quality of the trials included in analyses. Varker et al. (2021) found that only 7% of clinical trials operationally define dropout. Although CPT, PE, and EMDR, widely recognized as the “gold‐standard” treatments for PTSD, may have the strongest available evidence base supporting their effectiveness for treating PTSD, their dropout rates raise the urgency with which evidence‐based adaptations to these treatments must be developed to improve completion rates (Najavits, 2015).

One possible adaptation might be to modify the frequency of sessions to improve retention rates in trauma‐focused therapies (Leithner et al., 2026). Early research suggested that trauma‐focused treatment delivered daily or nearly daily (i.e., “massed”) demonstrated noninferiority to standard delivery in cognitive therapy (Ehlers et al., 2014) and PE (Foa et al., 2018). Weinstein et al. (2023) found that individuals who elected to receive “intensive CPT” (i.e., delivered at a higher frequency than the standard weekly regimen) were twice as likely to complete treatment than those who received standard CPT. A notable limitation of this finding is the lack of randomization: Patients who elected to receive intensive CPT may have had higher levels of motivation to engage in and maintain treatment than those who opted for the less‐intensive regimen. Models of accelerated service delivery (Sherrill et al., 2024) appear to be a promising strategy for reducing dropout while maintaining effectiveness. Similarly, some research suggests that PE administered twice weekly may slightly reduce treatment dropout compared with weekly PE (Dell et al., 2023; Levinson et al., 2022; Peterson et al., 2023).

In their systematic review and meta‐analysis, Sciarrino et al. (2020) found that increasing the frequency of PE or CPT to more than two sessions per week could increase treatment response and completion rates. Although this review marked one of the first meta‐analytic investigations of treatment completion in trauma‐focused therapy, with a shocking 5.5% pooled dropout rate, conclusions were sobered by the inclusion of just two randomized controlled trials in a sample of 11 total studies.

The present study sought to extend previous research by (a) evaluating the statistical association between treatment frequency and completion among gold‐standard treatments, (b) examining the possible interaction of treatment frequency and treatment type, and (c) investigating how researchers operationalize treatment completion, across clinical trials of trauma‐focused therapy for PTSD delivered in an outpatient setting. Given that weekly delivery largely remains the conventional cadence in practice, we sought to investigate whether increasing treatment frequency resulted in a statistically significant (*p* < .05) increase in the odds of achieving treatment completion rates relative to weekly regimens. This dichotomous definition of treatment frequency was chosen to offer clinicians a clear, direct comparison between conventional delivery and a faster cadence of treatment. These findings will inform guidelines needed to facilitate improved data‐sharing, replicability, and cumulative science, with downstream improvements in clinicians’ ability to properly tailor treatment planning to improve treatment retention and outcomes.

## METHOD

### Data source and procedure

Data for this cross‐study comparison analysis were gathered from the PTSD Repository, publicly available through the National Center for PTSD (National Center for PTSD, n.d.; O'Neil et al., 2020). The PTSD Repository is an international repository of clinical trials investigating pharmacological and nonpharmacological treatments for PTSD and was created to “serve as a data source for future systematic reviews, meta‐analyses, or other cross‐study comparisons” (O'Neil et al., 2020, p. 411). Clinical trials recorded in the PTSD Repository were included if they were completed in the United States, reported data for both treatment frequency and completion rates, and exclusively investigated psychotherapeutic interventions for PTSD. Additionally, to minimize the potential influence of setting (e.g., inpatient, intensive outpatient) on treatment frequency and retention, trials must have occurred in an outpatient setting. To evaluate differences in frequency and completion rates across gold‐standard treatments, the primary analysis was restricted to arms that included CPT, PE, and EMDR delivery. The secondary analysis assessed overall reporting practices for defining treatment completion. This study involved a secondary analysis of publicly available, aggregated study‐level data. No individual participant‐level data or identifiable private information were accessed or analyzed. Accordingly, this study did not constitute human‐subjects research, as defined under 45 CFR 46.102, and institutional review board review and approval were not required.

### Measures

#### Treatment completion (dependent variable)

The primary dependent variable was treatment completion. Treatment completion was operationalized at the study‐arm level as the proportion of participants who completed psychotherapy, as reported in each trial. When necessary, reported percentages were converted to proportions (0–1 scale), and the number of completers per arm was derived by multiplying the number randomized by the completion proportion and rounding to the nearest whole number. Variations in how individual studies defined treatment completion were reviewed in the PTSD Repository as a secondary outcome (Table 2). Implications of variations in how completion is defined on the primary outcome are discussed.

#### Treatment characteristics (independent variables)

The primary independent variable for the analysis was treatment frequency, categorized as weekly or more than weekly. Covariates included therapy type (CPT, EMDR, PE), total treatment duration (number of sessions), potential interaction between treatment type and treatment frequency, mean participant age in each study arm, and study‐level risk of bias (low risk, some concern, high risk).

### Data analysis

Data analyses were completed using RStudio (Version 2024.12, Build 467). Study arm was selected as the unit of analysis due to the preponderance of comparative effectiveness trials included in the data repository. A mixed‐effects binomial regression model was employed at the arm‐level of analysis to evaluate potential predictors of treatment completion. A random intercept was added to account for clustering by trial.

Sensitivity analyses were conducted to evaluate model fit, with consistent directionality and magnitude of model findings, as well as a change of less than 25.0% in the incidence rate ratio (IRR) used as the threshold indicative of model robustness. The analytic model, including all predictors and outcomes, was fully specified a priori, and no post hoc modifications to the model were made following estimation.

## RESULTS

A flowchart for study selection is provided in Figure 1. In total, 558 psychotherapy‐only intervention study arms were available in the Study Intervention database of the PTSD Repository. A total of 27 arms were excluded due to missing completion and frequency data after two attempts to reach study authors (see the Supplementary Materials for a list of studies excluded due to missing data). Staged interventions (e.g., daily‐to‐weekly, weekly‐to‐monthly) were excluded from the analysis due to underrepresentation. After applying all other selection criteria, a final sample size of 283 arms across 167 trials was obtained, with a subset of 84 arms (across 66 trials) of CPT, PE, or EMDR for the primary analysis. Characteristics of the study sample are provided in Table 1, and a list of all included studies is available in the Supplementary Materials.

**FIGURE 1:**
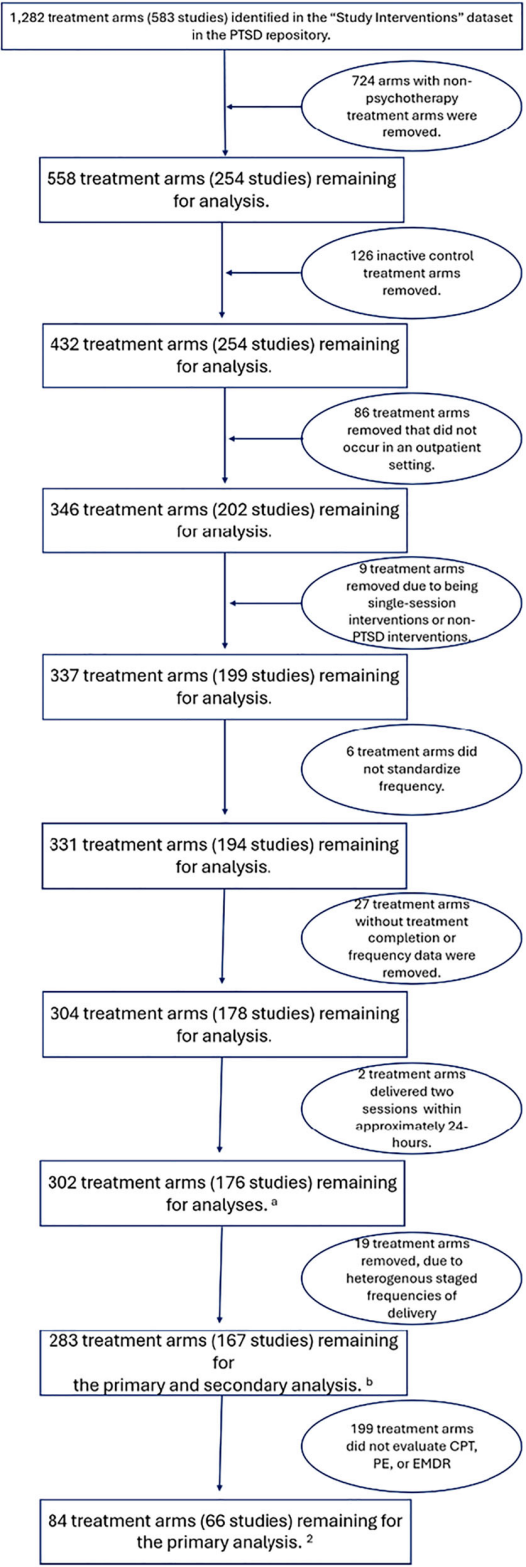
Flowchart of study selection *Note*: CPT = cognitive processing therapy; EMDR = eye movement desensitization and reprocessing; PE = prolonged exposure; PTSD = posttraumatic stress disorder. ^a^Used for the secondary analysis. ^b^Used for the primary analysis.

**TABLE 1:** Characteristics of the study arms

Variable	*Mdn*	IQR	*n*	%
Sample demographic characteristics				
Number of participants per arm	47	19–63		
Mean age of participants per arm (years)	38.9	33.8–44.1		
Female participants per arm (%)	56.2	10.9–91.6		
Military status of sample				
Active duty military			7	8.3
Community			39	46.4
Mixed			10	11.9
Veteran			25	29.8
N/A			3	3.6
Treatment completers per arm (%)	69.6	57.3–80.9		
Treatment characteristics				
Total treatment sessions per arm	12	9–12		
Treatment frequency				
Weekly			52	61.9
More than weekly			32	38.1
Treatment type				
Cognitive processing therapy			30	35.7
Prolonged exposure			35	41.7
Eye movement desensitization therapy			19	22.6

*Note*: IQR = interquartile range; N/A = nonapplicable.

**TABLE 2:** Variations in operationalization of treatment completion, across clinical trials of gold standard treatments^a^

Variable	CPT				PE				EMDR			
	Arm		Study		Arm		Study		Arm		Study	
	(*n =* 30)		(*n* = 25)		(*n* = 35)		(*n* = 30)		(*n* = 19)		(*n* = 16)	
	*n*	%	*n*	%	*n*	%	*n*	%	*n*	%	*n*	%
Completed or attended treatment (not further defined)	5	16.7	5	20.0	8	22.9	8	26.7	6	31.6	6	37.5
Completed treatment (not further defined)	5		5		6		6		6		6	
Completed/received full intervention (not further defined)					2		2					
Completed all sessions or treatment course	12	40.0	10	40.0	11	31.4	9	30.0	5	26.3	3	18.8
Completed all sessions	7		6		6		6		1		1	
Completed all sessions and possibly more	5		4		4		2		4		2	
Completed entire treatment course					1		1					
Attended all sessions												
Completed at least 50% of treatment sessions	11	36.7	8	32.0	7	20.0	5	16.7	2	10.5	2	12.5
Attended 80%–89% of sessions	4		3		2		2					
Attended 70%–79% of sessions	5		3						1		1	
Attended 60%–69% of sessions	1		1		3		2					
Attended 50%–59% of sessions	1		1		2		1		1		1	
Did not discontinue or drop out of intervention/treatment	1	3.3	1	4.0	1	2.9	1	3.3	3	15.8	3	18.8
Did not discontinue intervention	1		1									
Did not drop out					1		1		3		3	
Did not drop out (during waiting period)												
Did not miss last treatment session												
Clinical improvement					4	11.4	3	10.0				
> 50% or earlier with therapist agreement of noticeable improvement												
Completed treatment or > 75% with 70% reduced MPSS‐R score					2		1					
Completed treatment or PCL score < 16 for > 3 weeks					1		1					
> 83% or earlier with “sufficient improvement” and agreement with therapist					1		1					
Other									2	10.5	1	6.3
Attended ≥ 25% of all therapy sessions												
Completed all individual exposure sessions and 75% of group sessions												
Participated in treatment												
Received 2nd (final) treatment session									2		1	
Missing	1	3.3	1	4.0	4	11.4	4	13.3	1	5.3	1	6.3

*Note*: CPT = cognitive processing therapy; EMDR = eye movement desensitization and reprocessing; PTSD = posttraumatic stress disorder; MPSS‐R = Modified PTSD Symptom Scale–Self‐Report; PCL = PTSD Checklist; PE = prolonged exposure;

^a^ For a table of operationalized definitions of treatment completion across all 283 studies reviewed in the secondary analysis, see Supplementary Table S1.

No overdispersion or zero‐inflation was evidenced in the dataset. “Leave‐one‐out” sensitivity analyses showed that estimates for treatment frequency and its interactions varied modestly across deletions, as expected in a heterogeneous meta‐analytic dataset. No single study exerted undue influence on the directionality or magnitude of the findings, suggesting acceptable model robustness for the present analysis. Nontrivial study‐level clustering was observed, ICC = .07, justifying multilevel modeling. Sensitivity analyses performed for country‐level variation, military status, or dichotomized treatment completion definitions did not alter model performance or directionality (Supplementary Table S2).

### Treatment frequency and completion of CPT, EMDR, and PE

Sensitivity analyses were conducted to dichotomize the data into weekly treatment regimens (*n* = 52) and arms surpassing weekly treatment regimens (*n =* 31). Treatment types included CPT (*n* = 30), PE (*n* = 35), and EMDR (*n* = 19). The main‐effects model is illustrated in Table 1. Increasing treatment frequency from weekly to more than weekly did not demonstrate a detectable effect, odds ratio (*OR*) = 0.947, 95% confidence interval (CI) [0.63, 1.41]. Regarding treatment type, PE demonstrated modest reductions in the odds of completion compared to CPT arms, *OR* = 0.73, 95% CI [0.62, 0.86], whereas the odds of completing treatment were twice as high for EMDR, *OR* = 2.44, 95% CI [1.82, 3.27]. Risk of bias and age did not significantly shift the odds of completion. The marginal effect of treatment duration, while statistically significant, is unlikely to have exerted any meaningful effect on treatment completion, *OR* = 0.93, 95% CI [0.91, 0.97].

Although increasing frequency from weekly to more than weekly did not demonstrate a detectable effect on overall completion, an interaction effect between treatment type and frequency was observed (Figure 2). Specifically, shifting frequency from weekly to a faster cadence doubled the odds of completing PE, compared to CPT, *OR* = 2.15, 95% CI [1.55, 2.99]. Increasing EMDR did not demonstrate a statistically significant interaction effect, *OR =* 1.61, 95% CI [0.96, 2.71]. Estimated marginal means were calculated to estimate associations between frequency and completion rates, stratified by treatment type (Figure 3); this analysis supports the model's finding that increasing treatment frequency improves the odds of PE completion. Additionally, within EMDR, the predicted mean for completion rose by approximately 7% when delivered more than weekly, *p* = .023.

**FIGURE 2:**
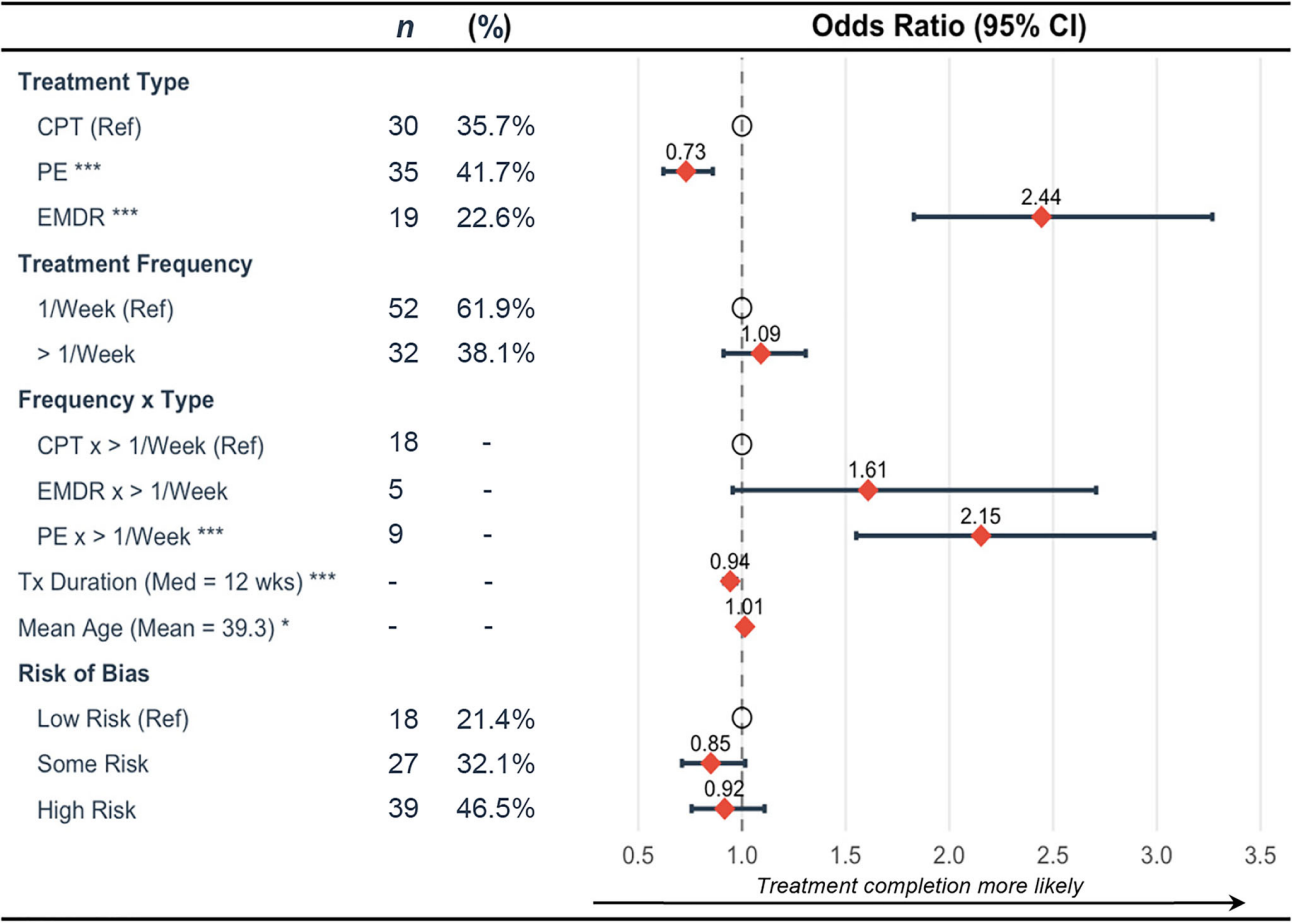
Forest plot of odds ratios for main effects on treatment completion *Note*: CI = confidence interval; CPT = cognitive processing therapy; EMDR = eye movement desensitization and reprocessing; PE = prolonged exposure; Ref = reference category; Tx = treatment. *p < .05. **p < .01. ***p < .001.

**FIGURE 3:**
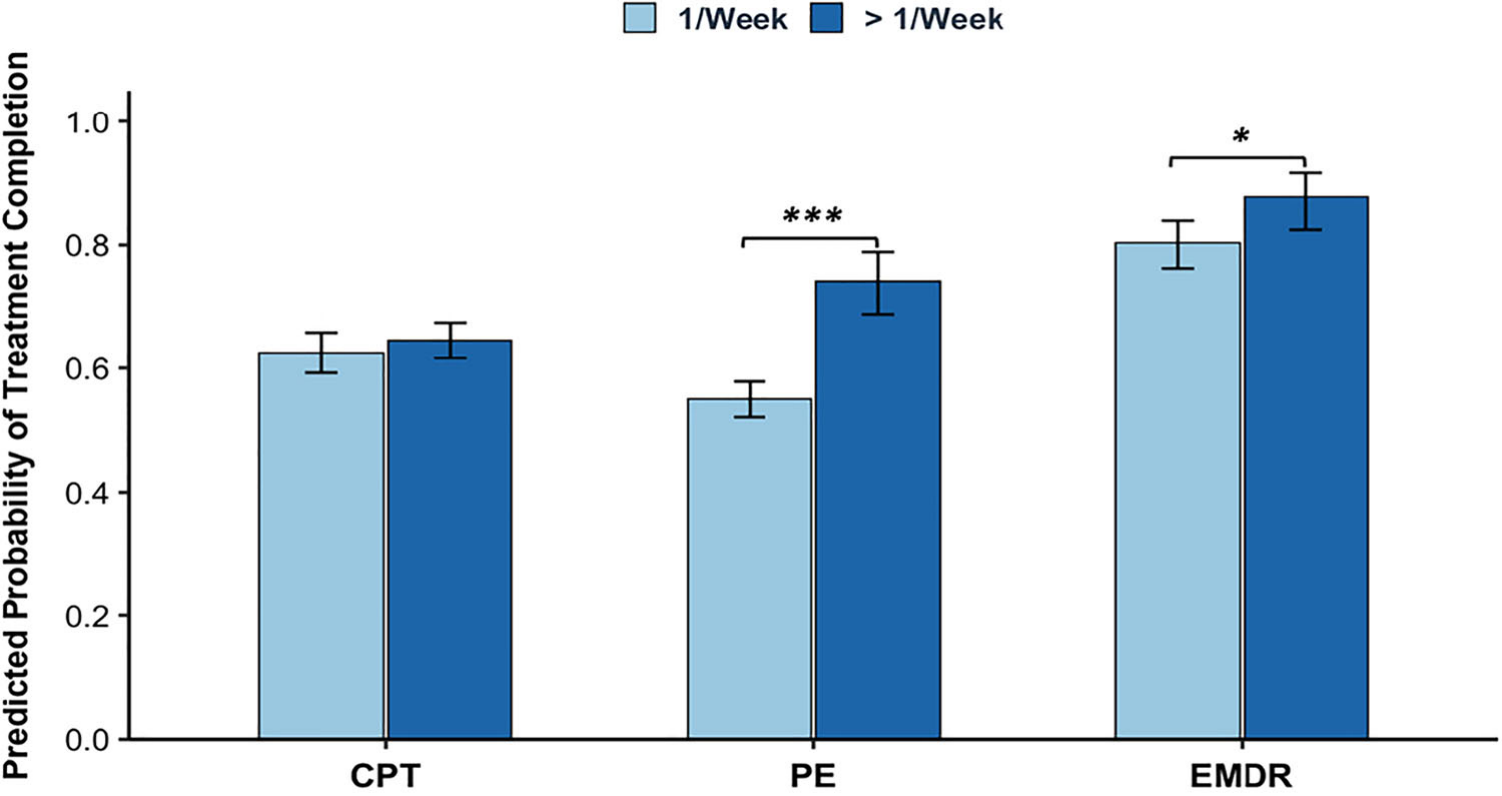
Interaction effects of treatment type and treatment frequency on treatment completion *Note*: Error bars represent 95% confidence intervals. The *p* values indicate the statistical significance of the difference between frequency groups (1/week vs. > 1/week) within each treatment type. These estimates are derived from the Treatment Type x Frequency interaction term in the multivariable generalized linear mixed model, adjusted for age, treatment duration, and risk of bias. CPT = cognitive processing therapy; EMDR = eye movement desensitization and reprocessing; PE = prolonged exposure. *p < .05. ***p < .001.

### Trends in reporting treatment completion

Across 283 study arms and 167 studies, definitions of treatment completion were classified into six categories. After at least two attempts to reach study authors for missing data, only 27 arms (9.5%) were excluded due to missing completion and/or frequency data (Supplementary Materials). The most common definition of treatment completion included some form of “complete treatment” (*n* = 106, 37.5%), without any additional information. A total of 63 treatment arms (22.3%) defined completion as completing all sessions, whereas another 21.2% of the included study arms allowed partial completion to be defined as treatment completion.

## DISCUSSION

Psychotherapy, particularly trauma‐focused treatments such as PE, EMDR, and CPT, remains the recommended first‐line treatment for PTSD, although suboptimal treatment completion rates remain a concern. The possibility of modifying treatment frequency to enhance retention has face validity, with previous research suggesting its viability. Although prior research suggests modifications to treatment frequency might enhance the feasibility of treatment completion, the present analysis suggests that, in aggregate, the benefits of modifying frequency appear to improve completion for PE while only modestly improving predicted completion means for EMDR and offering no detectable effect for CPT. For EMDR, the interaction estimate was not statistically significant, *OR* = 1.61, 95% CI [0.95, 2.70]. Estimated marginal means demonstrated a 19.2% higher probability of completing treatment when PE was delivered more than weekly, *p <* .001. These findings align with previous PE research suggesting this modality may benefit from a more intensive delivery frequency (Peterson et al., 2023). EMDR demonstrated a small (7.4%) increase in the probability of treatment completion when delivered more than weekly, *p* = .023, although this finding should be interpreted cautiously due to the limited number of EMDR arms included in the analysis. The lack of any statistically significant effect of frequency on completion for CPT or EMDR suggests that flexibility in the frequency of delivering these interventions may reasonably be left up to clinician judgment, although limited sample size, especially in EMDR treatment arms, might have precluded detection of significance.

We also found that current methods for operationalizing and reporting treatment completion are often too vague and/or inconsistent to facilitate more robust cumulative science. Although many of these definitions may represent similar notions of treatment completion (e.g., “completed treatment,” “did not discontinue intervention”), these definitions also create ambiguity that obfuscated conclusions. For instance, was “completed treatment” defined as attending all sessions or achieving a clinically significant reduction in symptoms to justify early termination? Similarly, was the definition “did not discontinue” inclusive of cases in which early termination occurred, or did this definition only reflect participants who dropped out of the study? The transparent defining and reporting of operationalized constructs such as treatment completion is integral to the successful implementation of evidence‐based interventions. Professional organizations (e.g., ISTSS), grant funders (NIH, DoD), and academic journals should strongly encourage consistency in operationalizing and reporting outcomes in clinical trials and quality improvement studies of PTSD interventions and develop and disseminate guidelines for what data are expected in publications for clinical trials, how such data should be defined, and how the data should be reported. Such strategies harbor the potential to enhance the possibility of a continuous learning cycle to improve treatment for PTSD.

Several limitations exist in the present analysis. Primarily, limitations in the sample size (84 “Tier 1 treatment” arms in the primary analysis) constrained model size to a handful of covariates. A smaller‐than‐anticipated sample size for the Treatment Frequency x Treatment Type interaction term should be interpreted as exploratory and warrants additional investigation. Additionally, across all 283 psychotherapy arms that met all other eligibility criteria, six distinct categories of definitions for treatment completion were identified, with “completed treatment (not further defined)” appearing with the highest frequency (37.5%). Although some variation in how completion is defined might be expected (e.g., eight sessions vs. 12 sessions), across various psychotherapy modalities, even standardizing completion by the percentage of sessions resulted in substantial heterogeneity. Subtle but potentially important differentiations in definitions, such as “completed treatment” and “completed all treatment sessions,” preclude robust comparisons.

Additionally, over half of the arms included in this analysis evaluated weekly treatment regimens; more frequent regimens were less common and heterogeneous (e.g., twice per week, daily), precluding a deeper analysis of treatment completion rates across these unique regimens that included more than one session per week. As a result, the model used in this study demonstrated an acceptable, but still mild, sensitivity to leave‐one‐out analyses, suggesting that additional arms—especially those assessing interventions delivered more than once a week—would increase model robustness in the future. This may be particularly important in allowing for future comparisons between weekly, biweekly, and “massed” models of PTSD care, which typically consist of three or more sessions per week and have shown promising results with respect to dropout and effectiveness (Dell et al., 2023; Holliday et al., 2025; Peterson et al., 2023).

Many future analytic directions are possible and needed in this space. The present paper focused on EMDR, CPT, and PE; future research might investigate the associations between frequency and completion in other evidence‐based modalities. Similarly, as interventions with staggered frequencies (e.g., daily for 1 week, then weekly thereafter) continue to build an evidence base, future analyses might compare completion rates between interventions with staggered or static frequencies. Evaluating the association between clinical and completion outcomes will also be critical, including how variations in outcome are measured and operationalized, and how such variation may moderate that association. Additionally, future moderator analyses might identify factors that moderate the association between treatment frequency and the likelihood of dropout; an adaptive approach that titrates frequency based on such moderators might facilitate optimization of treatment frequency to maximize the odds of completion.

However, such future research must incorporate shared, standardized definitions for core constructs, such as treatment completion, to promote replicable, cumulative science. Moreover, standards for operationalizing treatment completion should move beyond the realm of clinical trials to guide practicing clinicians, as well as to enable rapid learning and continuous quality improvement. This is particularly germane to first‐line treatments like CPT, EMDR, and PE, which continue to demonstrate suboptimal rates of noncompletion in clinical research and practice.

To that end, there is ongoing debate regarding how treatment completion should be defined, such as treatment response (only used in 2.5% of studies included in this analysis) versus the total number of treatment sessions completed. If clinicians would like to use “clinical judgement” to distinguish early treatment completers from treatment noncompleters, standardization regarding how patients are clinically determined to be early completers will help guide best practices.

To our knowledge, the present analysis is the first to directly compare outpatient session frequency and completion rates for EMDR, PE, and CPT. Although treatment dropout is a well‐documented phenomenon in PTSD—even in treatments labeled as “gold‐standard”—substantial heterogeneity in how researchers and practitioners define “treatment completion” precludes truly robust meta‐analyses on how to enhance completion outcomes. Therefore, PTSD leadership, funding bodies, and academic journals should strongly encourage, if not require, consistency in operationalizing and reporting outcomes in clinical trials and quality improvement studies of PTSD treatment, with particular emphasis on the defining, measuring, and reporting of treatment completion.

## AUTHOR CONTRIBUTIONS

**David Horton**: conceptualization, investigation, writing–original draft, methodology, validation, visualization, writing–review and editing, formal analysis, project administration, supervision, resources. **Gan Shi**: writing–review and editing, visualization, formal analysis, validation. **Matthew Davis**: methodology, writing–review and editing, validation. **Peter Grau**: validation, methodology, writing–review and editing. **Amy Kilbourne**: writing–review and editing, validation, supervision.

## AUTHOR NOTE

Amy Kilbourne is the Acting Deputy Chief Research and Development Officer for Investigators, Scientific Review, and Management for the U.S. Department of Veterans Affairs (VA) Office of Research and Development. The database accessed for this analysis was obtained from a repository maintained by the VA. The views expressed are those of the authors and do not necessarily represent the views of the VA or other government agency.

The authors wish to acknowledge Dr. Andrew Grogan‐Kaylor for his feedback on the mixed effects model used in this analysis, as well as Dr. Tanner Caverly for providing conceptual and editorial feedback on the manuscript.

## OPEN PRACTICES STATEMENT

Data were collected from the publicly available National PTSD Repository database, which can be accessed at https://www.ptsd.va.gov/ptsdrepository/index.asp

## Supporting information



Supplementary Table S1 Multivariable Mixed‐Effects Logistic Regression Controlling for Country of Study
Table S2. Multivariable Mixed‐Effects Logistic Regression Controlling for Military Status
Table S3. Multivariable Mixed‐Effects Logistic Regression Controlling for Definition of Treatment Completion
Table S4. Operationalized Treatment Completion Definitions for All Studies in Secondary Aim
