# Supplementary material for: Pacing in posttraumatic stress disorder therapy: A meta‐regression of treatment frequency and completion rates
Source: J Trauma Stress. 2026 Jul 26;39(5):775–84. doi: 10.1002/jts.70111 (PMC13639215; doi:10.1002/jts.70111)
Supplement: Supplementary file 1 [file JTS-39-775-s001.docx]

**Appendix 2: Sensitivity Analyses & Operationalized Treatment Completion Definitions**

*Supplementary Table S1 Multivariable Mixed-Effects Logistic Regression Controlling for Country of Study*

|  | *Odds Ratio (OR)* | *95% CI* | *P-value* |
| --- | --- | --- | --- |
| Frequency (> 1 Session/Week)ª | 1.05 | 0.73 – 1.53 | 0.784 |
| Treatment Type (ref: CPT) |  |  |  |
| EMDR | 1.38 | 0.77 – 2.49 | 0.282 |
| PE | 0.67 | 0.53 – 0.84 | < .001*** |
| Covariates |  |  |  |
| Treatment Duration (Weeks) | 0.92 | 0.87 – 0.98 | 0.008** |
| Age (Mean) | 1.02 | 1.00 – 1.04 | 0.123 |
| Risk of Bias: Lowᵇ | 0.96 | 0.66 – 1.39 | 0.827 |
| Risk of Bias: Some Concernsᵇ | 1.11 | 0.79 – 1.56 | 0.553 |
| Interactions |  |  |  |
| EMDR × Frequency (>1/Week) | 1.44 | 0.61 – 3.44 | 0.407 |
| PE × Frequency (>1/Week) | 2.16 | 1.31 – 3.59 | 0.003** |
| *Abbreviations*: CPT, Cognitive Processing Therapy; PE, Prolonged Exposure; EMDR, Eye Movement Desensitization Therapy; CI, Confidence Interval. *Note*. The Intraclass Correlation Coefficient (ICC) for the country effect is 0.067. ª Reference category is 1 Session/Week. ᵇ Reference category is High Risk of Bias. | | | |

*Table S2. Multivariable Mixed-Effects Logistic Regression Controlling for Military Status*

|  | *Odds Ratio (OR)* | *95% CI* | *P-value* |
| --- | --- | --- | --- |
| Frequency (> 1 Session/Week)ª | 1.02 | 0.68 – 1.53 | 0.920 |
| Treatment Type (ref: CPT) |  |  |  |
| EMDR | 1.71 | 1.01 – 2.91 | 0.046* |
| PE | 0.67 | 0.53 – 0.84 | < .001*** |
| Covariates |  |  |  |
| Treatment Duration (Weeks) | 0.92 | 0.86 – 0.99 | 0.023* |
| Age (Mean) | 1.04 | 1.01 – 1.08 | 0.013* |
| Risk of Bias: Lowᵇ | 0.89 | 0.58 – 1.37 | 0.595 |
| Risk of Bias: Some Concernsᵇ | 1.16 | 0.79 – 1.71 | 0.447 |
| Military Status (ref: Active Duty) |  |  |  |
| Community | 1.44 | 0.80 – 2.60 | 0.221 |
| Mixed | 0.75 | 0.36 – 1.54 | 0.431 |
| Veteran | 0.73 | 0.37 – 1.47 | 0.379 |
| **Interactions** |  |  |  |
| EMDR × Frequency (>1/Week) | 1.50 | 0.59 – 3.79 | 0.391 |
| PE × Frequency (>1/Week) | 1.98 | 1.13 – 3.46 | 0.016* |
| *Abbreviations*: CPT, Cognitive Processing Therapy; PE, Prolonged Exposure; EMDR, Eye Movement Desensitization Therapy; CI, Confidence Interval. *Note*. The Intraclass Correlation Coefficient (ICC) is 0.055. ª Reference category is 1 Session/Week. ᵇ Reference category is High Risk of Bias. | | | |

*Table S3. Multivariable Mixed-Effects Logistic Regression Controlling for Definition of Treatment Completion*

|  | *Odds Ratio (OR)* | *95% CI* | *P-value* |
| --- | --- | --- | --- |
| Frequency (> 1 Session/Week)ª | 0.97 | 0.66 – 1.41 | 0.856 |
| Treatment Type (ref: CPT) |  |  |  |
| EMDR | 2.04 | 1.25 – 3.32 | 0.004** |
| PE | 0.69 | 0.55 – 0.86 | 0.001*** |
| Covariates |  |  |  |
| Treatment Duration (Weeks) | 0.94 | 0.89 – 1.00 | 0.042* |
| Age (Mean) | 1.03 | 1.00 – 1.05 | 0.029* |
| Risk of Bias: Lowᵇ | 0.98 | 0.67 – 1.44 | 0.921 |
| Risk of Bias: Some Concernsᵇ | 1.19 | 0.82 – 1.71 | 0.357 |
| Treatment Completionᵈ |  |  |  |
| Complete Some Sessions | 0.67 | 0.48 – 0.95 | 0.025* |
| Did not drop out or discontinue | 1.29 | 0.70 – 2.39 | 0.412 |
| Interactions |  |  |  |
| EMDR × Frequency (>1/Week) | 1.42 | 0.61 – 3.30 | 0.414 |
| PE × Frequency (>1/Week) | 2.06 | 1.22 – 3.46 | 0.007** |
| *Abbreviations*: CPT, Cognitive Processing Therapy; PE, Prolonged Exposure; EMDR, Eye Movement Desensitization Therapy; CI, Confidence Interval. *Note*. The Intraclass Correlation Coefficient (ICC) is 0.056. ª Reference category is 1 Session/Week. ᵇ Reference category is High Risk of Bias. ᵈ Protocol Completion (Ref) includes studies requiring attendance of all sessions, the full intervention, or adherence to the full protocol. Complete Some Sessions includes studies defining completion by a specific number of sessions (e.g., >6) or a percentage (e.g., >75%). Did not drop out or discontinue includes studies defining completion as not dropping out or discontinuing treatment. | | | |

*Table S4. Operationalized Treatment Completion Definitions for All Studies in Secondary Aim*

| **Categories of Treatment Definitions, *n* (%)** | **Arm** |  | **Study** |  |
| --- | --- | --- | --- | --- |
| *SubCategories of Definitions, n* | *N*=283 |  | *N*=167 **^a^** |  |
|  | *n* | % | *n* | % |
| **Completed or attended treatment (not further defined)** | **106** | **37.5** | **66** | **39.5** |
| *Completed Treatment (not further defined)* | *76* |  | *52* |  |
| *Completed/Received Full Intervention (not further defined)* | *30* |  | *14* |  |
| **Completed all sessions or treatment course** | **63** | **22.3** | **42 (25.1)** |  |
| *Completed All Sessions* | *37* |  | *24* |  |
| *Completed All Sessions and Possibly More* | *14* |  | *8* |  |
| *Completed Entire Treatment Course* | *7* |  | *6* |  |
| *Attended All Sessions* | *5* |  | *4* |  |
| **Completed at Least 50% of Treatment Sessions** | **60** | **21.2** | **38** | **22.8** |
| *Attended 80-89% of Sessions* | *19* |  | *10* |  |
| *Attended 70-79% of Sessions* | *19* |  | *13* |  |
| *Attended 60-69% of Sessions* | *15* |  | *10* |  |
| *Attended 50-59% of Sessions* | *7* |  | *5* |  |
| **Did not discontinue or drop out of intervention/treatment** | **18** | **6.4** | **11** | **6.6** |
| *Did not discontinue intervention* | *5* |  | *3* |  |
| *Did not drop out* | *10* |  | *6* |  |
| *Did not drop out (during waiting period)* | *1* |  | *1* |  |
| *Did not miss last treatment session* | *2* |  | *1* |  |
| **Clinical Improvement** | **7** | **2.5** | **4** | **2.4** |
| *>50% or earlier with Therapist agreement of noticeable improvement* | *1* |  | *1* |  |
| *Completed Treatment or >75% with 70% reduced score on MPSS-R* | *2* |  | *1* |  |
| *Completed Treatment or PCL <16 for >3 weeks* | *2* |  | *1* |  |
| *>83% or earlier with "sufficient improvement" and agreement with therapist* | *2* |  | *1* |  |
| **Other** | **6** | **2.1** | **4** | **2.4** |
| *Attended ≥25% of all therapy sessions* | *1* |  | *1* |  |
| *Completed all individual exposure sessions and 75% of group sessions* | *2* |  | *1* |  |
| *Participated in treatment* | *1* |  | *1* |  |
| *Received 2nd (final) treatment session* | *2* |  | *1* |  |
| **Missing** | **23** | **8.1** | **14** | **8.4** |

^a.^ While most studies included the same definitions of treatment completion, across arms, some studies included different definitions between arms. As a result, the values in this column do not add up to the total number of studies (167), as some studies may be included under multiple categories.
